# Energy expenditure and indirect calorimetry in critical illness and convalescence: current evidence and practical considerations

**DOI:** 10.1186/s40560-021-00524-0

**Published:** 2021-01-12

**Authors:** Hanneke Pierre Franciscus Xaverius Moonen, Karin Josephina Hubertina Beckers, Arthur Raymond Hubert van Zanten

**Affiliations:** 1 Department of Intensive Care Medicine, Gelderse Vallei Hospital, Willy Brandtlaan 10, 6716 RP Ede, The Netherlands; 2 Division of Human Nutrition and Health, Wageningen University & Research, HELIX (Building 124), Stippeneng 4, 6708 WE Wageningen, The Netherlands

**Keywords:** Energy expenditure (EE), Indirect calorimetry (IC), Resting energy expenditure (REE), Critical illness, Intensive care unit (ICU), Metabolism

## Abstract

The use of indirect calorimetry is strongly recommended to guide nutrition therapy in critically ill patients, preventing the detrimental effects of under- and overfeeding. However, the course of energy expenditure is complex, and clinical studies on indirect calorimetry during critical illness and convalescence are scarce. Energy expenditure is influenced by many individual and iatrogenic factors and different metabolic phases of critical illness and convalescence. In the first days, energy production from endogenous sources appears to be increased due to a catabolic state and is likely near-sufficient to meet energy requirements. Full nutrition support in this phase may lead to overfeeding as exogenous nutrition cannot abolish this endogenous energy production, and mitochondria are unable to process the excess substrate. However, energy expenditure is reported to increase hereafter and is still shown to be elevated 3 weeks after ICU admission, when endogenous energy production is reduced, and exogenous nutrition support is indispensable. Indirect calorimetry is the gold standard for bedside calculation of energy expenditure. However, the superiority of IC-guided nutritional therapy has not yet been unequivocally proven in clinical trials and many practical aspects and pitfalls should be taken into account when measuring energy expenditure in critically ill patients. Furthermore, the contribution of endogenously produced energy cannot be measured. Nevertheless, routine use of indirect calorimetry to aid personalized nutrition has strong potential to improve nutritional status and consequently, the long-term outcome of critically ill patients.

## Background

The optimal quantity and timing of nutrition support for critically ill patients has long been debated. In the past, nutrition guidelines supported early aggressive feeding to meet estimated energy expenditure (EE), aimed at the prevention of malnutrition and muscle loss. However, clinical studies have failed to prove an unequivocal benefit of early high-dose nutrition support, and several prospective randomized clinical trials showed significant harm, including increased hyperglycemia, hepatic steatosis, and mortality [1–5]. In contrast, undernourishment is also common in ICU and post-ICU patients due to both prescription inadequacy and failure to reach the nutrition target [6–12]. A negative energy balance in critically ill patients is associated with increased morbidity, including increased length of hospital stay, infections, organ failure, prolonged mechanical ventilation, and even mortality [2, 13]. Although there is a clear understanding that over- and underfeeding are associated with worse outcome, optimization of nutrition support is impeded by a lack of insight into the variable nutritional needs of critically ill patients during ICU stay and convalescence, both on a group and individual level [1, 8, 14]. The available evidence indicates numerous factors that may lead to significant daily variations in EE in and between critically ill patients [1, 15, 16]. Therefore, individualized real-time nutrition therapy is the next step toward optimal patientcare [1, 15, 17–21]. Indirect calorimetry (IC) is considered the gold standard to measure caloric needs in critically ill patients at bedside, and its use has been strongly recommended by the recent European Society for Clinical Nutrition and Metabolism (ESPEN) and American Society for Parenteral and Enteral Nutrition (ASPEN) guidelines [1, 16, 18, 22].

This narrative review aims to provide a detailed summary of current evidence on the course of energy expenditure and the use of IC in critically ill patients in the ICU and during the post-ICU hospital stay. We include practical aspects of the use of IC and implications for nutrition therapy.

## Energy expenditure

**Fig. 1:**
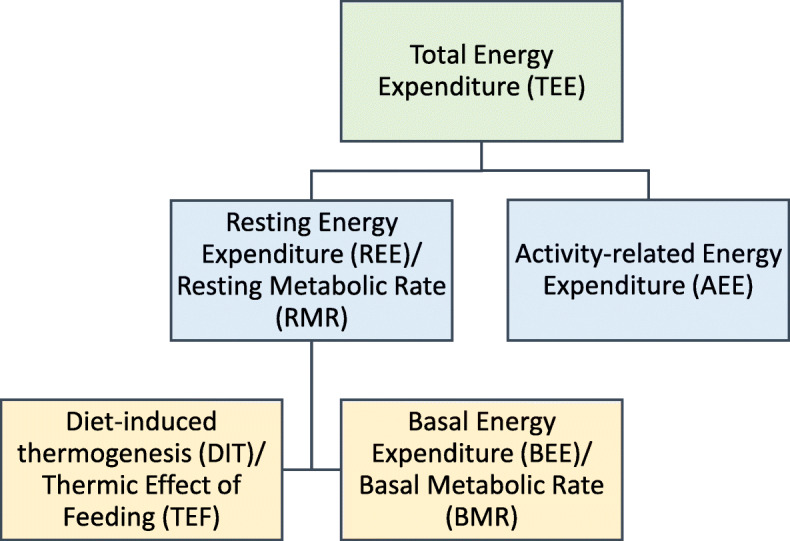
Components of energy expenditure

Total energy expenditure (TEE) is defined as the total amount of energy humans need to function. TEE can be subdivided into basal energy expenditure (BEE, or basal metabolic rate; BMR), diet-induced thermogenesis (DIT, or thermic effect of feeding; TEF), and physical activity-related energy expenditure (AEE). BEE and DIT combined, represent the resting energy expenditure (REE, or resting metabolic rate; RMR), which is defined as all energy requirements involved in the body’s basal metabolism to maintain vital functions while inactive (Fig. 1) [23–25]. REE can be measured by IC and in sedentary, healthy subjects, accounts for about two-thirds of TEE [23]. In critically ill patients, REE will closely reflect TEE because of minimal physical activity [8, 19].

### Energy expenditure during critical illness

Metabolic response to critical illness is complex and has been a subject of research and debate for decades [26].

#### Historical concepts

**Fig. 2:**
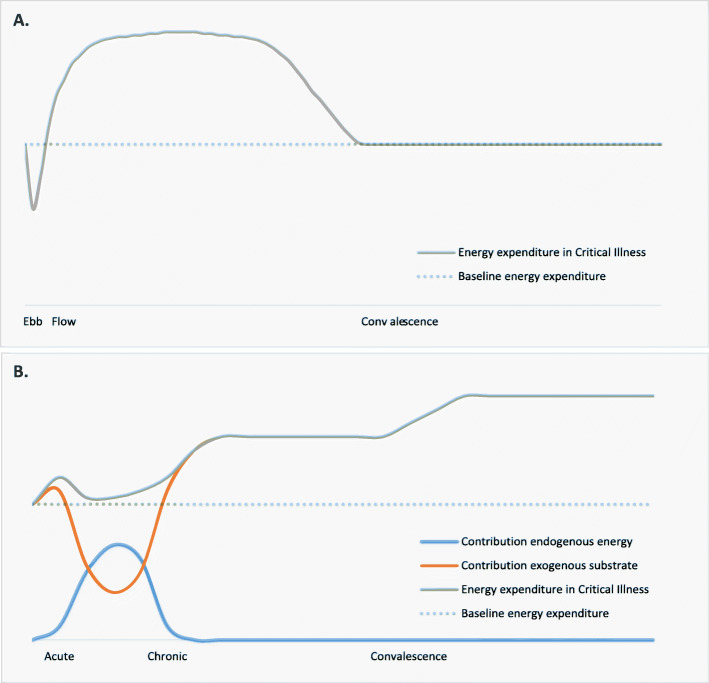
Progressing concepts of energy expenditure in critical illness. **a** Historical concept of energy expenditure in critical illness. **b** Current understanding of energy expenditure in critical illness and the contribution of various energy sources

In 1942, Sir Cuthbertson, described the metabolic response to traumatic stress as occurring in an ebb phase and a flow phase (Fig. 2) [26, 27]. The ebb phase lasted minutes to hours after the initial insult and was thought to be characterized by a decline in body temperature and oxygen consumption, aimed at reducing posttraumatic energy depletion [26]. After this brief phase of hypometabolism, Sir Cuthbertson and others recognized a significant increase, or “flow,” in metabolism, called traumatic inflammation, or hypermetabolism [28–31]. Hypermetabolism was thought to result from persistent catabolism, the systemic breakdown of lean tissue mass, and a rise in O_2_ consumption to produce endogenous energy substrates to meet the high energy requirements during critical illness [1, 2]. This increased catabolism leads to depletion of lean body mass, a syndrome which has been referred to as “autocannibalism” and feedings strategies were aimed at halting this process by satisfying the metabolic flow with substrate. The hypermetabolic phase was thought to end when the healing process began, with metabolism then reverting to the anabolic state [32].

#### Current understanding

Cuthbertson’s theory is still frequently cited; however, clinical trials have failed to identify a clear course of energy expenditure in all critically ill patients [33]. In addition, early aggressive feeding strategies have not had the desired and expected effect. The reality appears more complex and omnifarious than the theory.

The described ebb phase has not been clearly identified in vivo, and its clinical relevance is debatable because of its briefness. Besides, there is usually, and logically, an emphasis on hemodynamic, rather than metabolic stabilization and nutrition support during this phase of critical illness [34]. In line with the flow theory, it is known that the release of catabolic hormones such as norepinephrine, cortisol, and glucagon increases gluconeogenesis, glycogenolysis, mobilization of free fatty acids, and muscle proteolysis in the acute phase of critical illness [2, 17, 35, 36]. In addition, increased metabolism has been shown in several diseases, although patterns are highly variable, and the degree of increase from normal REE may reflect the severity of the metabolic response to the injury [1]. However, hypermetabolism does not always characterize the initial phase of critical illness, as several studies show that during the first days, oxygen consumption can fall to near-baseline levels [37–39] (Fig. 2). This phenomenon is hypothesized to be the result of a decrease in mitochondrial function as an adaptive strategy of metabolic hibernation to prevent cell death by energy substrate overloading at a time when mitochondria cannot keep up with energy demand [40]. In patients with sepsis, a reduced oxygen utilization by 22–42% was found, compared with healthy volunteers [41]. A higher REE in severe sepsis patients has been associated with higher mortality, further adding to the notion that the metabolic downregulation might be sometimes adaptive rather than a sign of malfunction [42].

Regardless of the rate of metabolism, some unique metabolic changes occur in the acute phase of critical illness, which helps explain the counterintuitive effects of early aggressive feeding. As metabolism is decreased, and catabolism has the upper hand, exogenous nutrient and insulin administration have been shown not to abolish endogenous glucose production [18, 43]. Therefore, the endogenous energy production is likely near sufficient to meet energy demand during this phase [30, 44]. As a result, full nutrition support may result in overfeeding [15, 17, 18]. To reflect this, current nutrition guidelines recommend a gradual increase in caloric intake during the first 3-5 days after ICU admission to avoid overfeeding [18, 22].

After several days, REE increases again, and as endogenous energy production is simultaneously reduced, the risk of underfeeding increases [15, 45, 46]. This might be considered the chronic metabolic phase of critical illness. An increase in REE has been demonstrated in both surgical and medical ICU patients, and a maximum REE is found around the ninth or tenth day after ICU admission [34, 38, 47–49]. Clinical data on the course of EE during the recovery or convalescence phase of critical illness is scarce and usually derived from studies with small sample size. When available, measured REE is still significantly elevated several weeks after ICU admission, as has been shown in burns, trauma, and sepsis patients, including very recently in COVID-19 [50–52]. However, in serial measurements in twelve patients during the post-ICU hospitalization period, Ridley et al. showed significant individual variability in measured EE [11]. During this phase, TEE is likely to once again increase above REE, due to increased physical and mental activity, as the focus of treatment is moved toward rehabilitation. Ideally, the patient enters a recovery phase with enhanced anabolism, requiring more substrate. In contrast, the persistent inflammation, immunosuppression, and catabolism syndrome (PICS) may arise in some [9, 18, 28]. Metabolically, PICS is characterized by a persistent catabolic state and hormonal disruption leading to anabolic resistance and inflammation-induced cachexia [53].

Thus, different metabolic phenotypes arguably require a different and individualized nutritional approach. In addition, many individual and iatrogenic factors might cause metabolic requirements to be highly variable among patients as well as over time, making them hard to predict [1, 51, 54]. Although they are not the same, regularly measured REE could be a useful proxy for real-time energy requirement in this vulnerable group of patients.

Table 1 summarizes factors influencing energy expenditure, including specifics of the underlying disease and its treatment, anthropometrics, nutritional status, (in)activity, and environment during and after critical illness.

**Table 1:** Factors affecting energy expenditure in critical illness

↑ Energy expenditure	↓ Energy expenditure
■ Caucasian ethnicity ■ Overfeeding ■ Physical exercise, agitation ■ ↑ Minute volume ■ Hyperthermia ■ Hyperthyroidism ■ Metabolic acidosis ■ Stress (cortisol, glucagon, norepinephrine) ■ Systemic inflammation, sepsis ■ Burns	■ Female sex ■ Older age ■ ↓ Lean body mass ■ Prolonged fasting, underfeeding ■ Paralysis, coma ■ ↓ Minute volume ■ Hypothermia ■ Hypothyroidism ■ Metabolic alkalosis ■ Medication: β-blockers, sedatives, muscle relaxants

Adapted from [1, 8, 19, 25, 55]. Symbols: ↑, increase(d); ↓, decrease(d)

## Indirect calorimetry

If and when the transition into different metabolic phases occurs in individual patients, it is still unidentifiable in clinical practice. Because of not only the high variability between patients, but also during the disease in the individual patient, regular measurements of EE by IC could provide a better target for nutrition therapy in the subsequent phases of disease and convalescence [17, 23].

### Indirect calorimetry in theory

IC measures respiratory gas exchange to estimate energy metabolism. On a cellular level, metabolism entails the production of adenosine triphosphate (ATP), with carbon dioxide (CO_2_) and water as by-products, by consuming oxygen (O_2_) and burning substrates such as glucose, free fatty acids, and amino acids. As the energy produced equals the energy consumed, IC measuring O_2_ consumption and CO_2_ production represents real-time energy metabolism [24, 30]. Direct calorimetry, in contrast, measures heat production and, therefore, energy production directly, but this method is not feasible in clinical practice, as it requires the patients to be measured inside an insulated chamber [23, 24].

IC determines REE by measuring oxygen consumption (VO_2_, in L/min) and carbon dioxide production (VCO_2_, in L/min) and subsequently calculates REE according to the adjusted Weir’s equation, based on the caloric values of the oxidation of 1 L of O_2_ metabolizing a fat and carbohydrate mixture [25, 56]. The original Weir equation includes urinary nitrogen measurement content representing protein oxidation. However, IC uses an adjusted version based on the Haldane transformation, which assumes that nitrogen is physiologically inert, and therefore, the volume of inspired nitrogen must equal the volume of expired nitrogen. This adjustment excludes the need for urinary measurements, which improves feasibility and introduces only a small error up to 1-2% occurs in final REE calculation [24, 25, 30, 57].

$$ \mathrm{REE}\ \left(\mathrm{kcal}/\mathrm{day}\right)=1.44\times \left(\left[{\mathrm{VO}}_2\ \left(\mathrm{mL}/\min \right)\times \kern0.37em 3.94\right]+\left[{\mathrm{VCO}}_2\ \left(\mathrm{mL}/\min \right)\times \kern0.37em 1.11\right]\right) $$

Furthermore, IC calculates a respiratory quotient (RQ) during measurement, i.e., the CO_2_-production to O_2_-consumption ratio [19, 25]:

$$ \mathrm{RQ}={\mathrm{VCO}}_2/{\mathrm{VO}}_2 $$

The RQ is an indicator of the composition of substrate use. It indicates which macronutrients are being metabolized, as different energy pathways are used. A human RQ of 1.0, 0.8, and 0.7 represents glucose, protein, and fat oxidation, respectively [23, 25, 30, 58]. The physiological range of the RQ is 0.67-1.3; therefore, it can also be used as a quality indicator of the measurement adequacy [59–61]. The approximate respiratory quotient of a mixed oral diet is 0.8.

### Indirect calorimetry devices

**Fig. 3:**
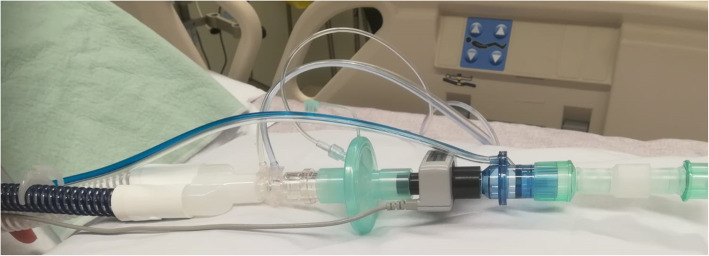
Insertion of a disposable flowmeter into the patient circuit of a mechanic-ventilation system (QNRG®, Cosmed, Italy)

**Fig. 4:**
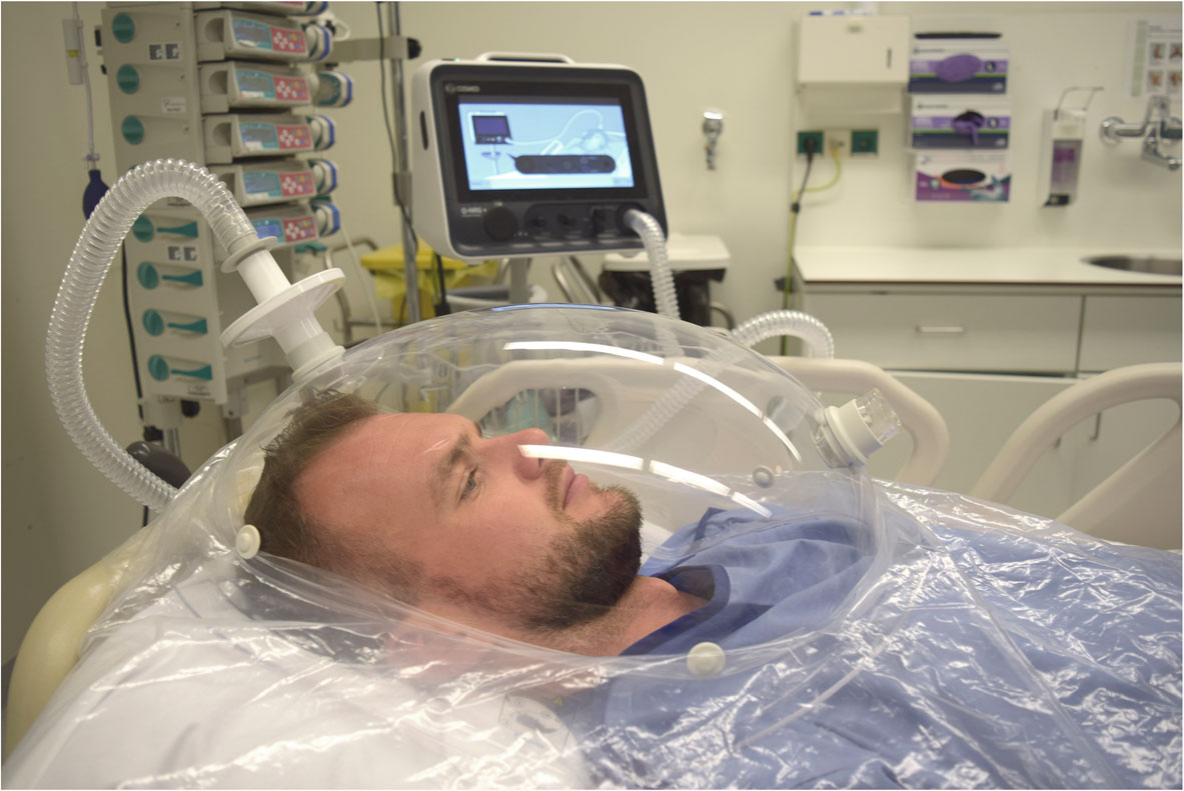
Use of a flow-dilution canopy hood to measure gas exchange in a spontaneously breathing patient (QNRG®, Cosmed, Italy)

IC measurements can be performed by using the ventilation circuit in mechanically ventilated patients for gas sampling, or by using a canopy hood or face mask in spontaneously breathing patients to analyze their in- and expired air (Figs. 3, 4) [19].

Many different devices are available [28]. The Deltatrac® (Datex, Finland) was the most validated metabolic monitor and frequently used until sales were discontinued [62–64]. Several other devices have made it to the market, each with its limitations. The Quark RMR® (Cosmed, Italy), E-COVX® (Datex-Ohmeda, Finland), CCM Express® (Medgraphics, USA), and Vmax® (Vyaire, USA) were shown to be equal or inferior to the Deltatrac on several aspects (Table 2) [16, 64–67]. In addition to these stand-alone devices, some mechanical ventilators have integrated IC functions, but its use has not yet been validated [63]. Lastly, some devices are small, and handheld, such as the Fitmate® (Cosmed, Italy) or MedGem® (Microlife, USA), but have not been validated in critically ill patients [1, 11, 65]. In order to overcome all disadvantages of the devices mentioned above. The Q-NRG® (Cosmed, Italy) has been developed by a task force of medical experts from the European Society of Intensive Care Medicine in the international calorimetry study initiative (ICALIC) project. It is the only device tested against mass spectrometry for accuracy during inspired fraction of oxygen (FiO_2_)—settings ranging from 0.21 to 0.70 and can be used in both mechanically ventilated and spontaneously breathing patients [16, 19, 62, 68].

**Table 2:** Overview of comparative studies of IC devices in mechanically ventilated patients [16, 64–67]

	Q-NRG®	Deltatrac®
**Deltatrac® (Datex, Finland)**	- (No) significant difference in measured REE [16] - Measurements using Q-NRG® significantly faster [16]	
**QUARK RMR®** **(Cosmed, Italy)**	- Significant difference in measured REE (*p* = 0.038) - Measurements using Q-NRG® significantly faster [16]	- No significant difference in mean REE (*p* = 0.166) - No significant differences EE, VCO_2_, and VO_2_ [66] - Significant difference in RQ (*p* < 0.0001) not favoring Deltatrac®, due to measurement values outside the physiological range [60] - Overestimation of VO_2_ and VCO_2_ by QUARK RMR® [67]
**Vmax® (Vyaire, USA)**	- Significant difference in measured REE (*p* < 0.001) - Measurements using Q-NRG® significantly faster [16]	- No significant difference in REE (*p* = 0.8) is not reliable enough in a clinical research setting [65]
**E-COVX® (Datex-Ohmeda, Finland)**	- No significant difference in measured REE (*p* = 0.165) - No significant difference in the duration of measurement	- Overestimation of VO_2_ and VCO_2_ by E-COVX® [67]
**CCM Express® (Medgraphics, USA)**		- Significant difference in mean REE (*p* < 0.0001) - Significant difference in RQ (*p* < 0.0001) - Significant differences in RQ and VO_2_ and VCO_2_ (*p* < 0.0001) [66]

### Obtaining reliable results

Even with an accurate device, many aspects have to be taken into account to ensure a reliable measurement and a valid interpretation of the results, especially when they consequently lead to an adjustment in nutrition therapy. Because an IC measurement is always a snapshot representation of a continuously changing metabolic state, it is essential to ensure as much of a steady state as possible and practical during the measurement procedure, so that momentary changes in the patient’s condition do not overly influence the interpretation of the baseline EE [7, 30, 69]. Furthermore, several conditions potentially influence the measurement itself by altering the gas flow [25].

#### Steady-state measurement

Many situations influence a patient’s steady-state (Table 1), and a patient should ideally not experience mental or physical stress, be physically active, or be fed shortly preceding or during the measurement [19]. We discuss several points of attention when performing IC in the intensive care setting.

The use of organ support devices for continuous renal replacement therapy (CRRT) and extracorporeal membrane oxygenation (ECMO) are everyday in the ICU setting. The influence of CRRT on REE is controversial [1, 70]. Theoretically, heparin-CRRT influences VCO_2_ measurements because an individual, unknown amount of CO_2_, is influenced by exogenous bicarbonate administration. It may thereby alter the outcome of IC measurement, although others reported that this difference might not be significant [1, 23, 71]. Continuous venovenous hemofiltration (CVVH) using citrate anticoagulation in the predilution mode, might affect REE in three ways. O_2_ and CO_2_ are exchanged in the CRRT circuit, theoretically affecting the Weir formula. Additionally, CRRT induces heat loss and immunologic activation. Lastly, calorie-containing molecules are exchanged within the filter, in addition to citrate itself [65]. The most recent study by Jonckheer et al. [72] in 10 critically ill ventilated patients treated with CVVH found that CO_2_ alterations due to CVVH are of no clinical importance, so no correction factor for REE is needed with or without CVVH. In contrast with previous recommendations suggesting initiation of IC only several hours after cessation of CVVH, Jonckheer et al. recommend performing IC measurements during CVVH, as CVVH does not seem to alter metabolism.

So far, guidelines lack specific recommendations on nutrition support for ECMO patients. Retrospective data show that underfeeding during ECMO is still prevalent, mainly due to interruptions and poor gastric motility [73]. Additionally, ECMO delivers O_2_ in addition to removing CO_2_, making reliable IC calculation and interpretation even more complicated [19]. De Waele et al. [74] proposed to insert consecutively obtained, individual IC measurements of the native and the artificial lung in the adjusted Weir equation to retrieve a measured REE composite as follows:

$$ {\mathrm{REE}}_{\mathrm{composite}}=1.44x\ \left(\left[3.94\times {\mathrm{VO}}_{2\mathrm{total}}\right]+\left[1.11\times {\mathrm{VCO}}_{2\mathrm{total}}\right]\right) $$

With VO_2total_ *=* VO_2native lung_ *+* VO_2ECMO_

     With VO_2native lung_ = VE × [Fi_O2_−Fi_O2_]

     And VO_2ECMO_ = [Fi_O2ECMO_ × VI_ECMO_]−[Fe_O2ECMO_ × VE_ECMO_]

And VCO_2total_ = VCO_2native lung_ + VCO_2ECMO_

     With VCO_2native lung_ = [Fe_CO2_ × VE_native lung_]−[Fi_CO2_ × VE_native lung_]

     And VCO_2ECMO_ = [Fe_CO2ECMO_ × VE_ECMO_]−[Fi_cO2ECMO_ × VE_ECMO_]

Wollersheim et al. [75] propose a similar equation combining traditional IC measurements of the native lung with calculations based on pre-membrane and post-membrane oxygenator blood gas analyses allowing for simultaneous measurements of lung and ECMO device.

$$ {\mathrm{VO}}_{2\mathrm{ECMO}}=\left[{\mathrm{O}}_{2\mathrm{BGApost}}-{\mathrm{O}}_{2\mathrm{BGApre}}\right]\times \mathrm{ECMO}\ \mathrm{blood}\ \mathrm{flow} $$

$$ {\mathrm{VCO}}_{2\mathrm{ECMO}}=\left[{\mathrm{CO}}_{2\mathrm{BGApre}}-{\mathrm{CO}}_{2\mathrm{BGApost}}\right]\times \mathrm{ECMO}\ \mathrm{blood}\ \mathrm{flow} $$

However, these small studies’ results require further validation in a larger ECMO patient cohort with different gas flow management [76].

At least 30-60 min preceding IC measurement, no medication alterations should be carried out [8, 63]. Sedatives and analgesics may cause reductions in VO_2_ and REE [4, 13]. Neuromuscular blocking agents also affect the EE, although the effect is small [8, 24, 28, 77, 78]. A recent study with continuous infusion of cisatracurium showed a significant reduction in EE measured with the VCO_2_ method, although the clinical relevance is presumed to be minor, and in most patients no reductions in caloric prescription are necessary [78]. Furthermore, the administration of vasopressors increases REE, whereas specific β-blockers are contradictory reported to decrease REE [8, 35, 77, 79]. However, the effect of low-dose cardio-specific β-blockers is negligible [75]. Consequently, IC measurements should be repeated as significant dose changes regarding levels of sedation or hemodynamic support are made [24].

From a mechanistic point of view, patients receiving bolus nutrition or orally fed patients should be fasted for at least 5 h before performing IC to obtain a stable measurement [63, 80]. However, this is often undesirable and unfeasible in clinical ICU practice [11, 63, 80]. In the case of continuous (par)enteral feeding, DIT has minimal effect on IC, if the infusion rate is not altered 1 h before or during measurement [8, 28].

Physical activity, including all body movements related to stress, such as agitation, seizures, shivering, invasive procedures, and unstable analgesia or sedation, can alter EE [19, 81]. Ideally, a patient should rest up to 20 min before IC takes place [76]. As this is often difficult, if not impossible to achieve in the ICU setting, these conditions may introduce error into the measurement if they do not resemble the patient’s steady state. Physiotherapy or active mobilization should be avoided 2 h before measurements. Endotracheal tube suction should be avoided within 20 min before and during measurements [63]. Ventilator settings should not be changed for 60 to 120 min before or during the IC measurement, as the patient needs to adjust to the new settings and therefore, might not be completely stable and at rest [8, 28, 77].

Body temperature variations of more than 1 °C before IC measurement, make results less reliable [28, 68, 78]. Some authors report an increase in REE caused by fever, whereas therapeutic hypothermia is associated with a decrease in REE; however, not all studies report similar findings [81].

#### Gas collection

The ventilation mode may unjustly influence measured EE by directly affecting the measured gas flow used for calculation [15, 82, 83]. As the device uses the amount of inspired and expired N_2_ as a control to define the amount of inspired and expired oxygen and carbon dioxide, the amount of N_2_ will be too low to get a reliable result, when the fraction of inspired oxygen is too high. Patients with an FiO_2_ > 0.6 cannot be measured accurately by most devices, although the Q-NRG can measure REE in mechanically ventilated patients with a FiO_2_ up to 0.7 [6, 28, 63]. Consequently, the use of nitric oxide also influences IC measurements [1, 23, 28]. Moreover, fast respiratory rates (> 35/min) lead to difficulty in the gas analysis [23, 30]. Patients with unspecified amounts of air leakage, such as an uncuffed tracheostomy cannula, endotracheal tube cuff leaks, tracheal-esophageal fistulae, subcutaneous emphysema, or chest tube drainages should be excluded from IC measurements, as the gas collection is unreliable [8, 19, 23]. Additionally, an error could be induced by air leakage, instable FiO_2_ or expiratory flow, compressed volume, and air trapping in patients with high positive end-expiratory pressure, i.e., PEEP> 10 cmH_2_O. Lastly, although the use of a canopy or hood makes measurements possible in spontaneously breathing patients with or without non-invasive ventilation, supplemental O_2_ cannot be adequately measured or incorporated into the equations [1, 84].

Although the precautions mentioned earlier aim to ensure a measured EE that reflects real caloric need as closely as possible, it is essential to realize that IC, unless performed continuously, always extrapolates measurements obtained from a short period and therefore never fully accounts for the variation of EE during 24 h [85]. IC measurements should ideally be repeated every 2 to 3 days if feasible and whenever a patient’s clinical condition or treatment changes significantly, thereby possibly influencing EE [17, 19, 86–88].

#### Practical considerations

No standardized protocol for performing IC is available [7]. However, it stands to reason that the metabolic monitor should be calibrated, connected, and operated correctly, and the technical ranges of the specific device should not be exceeded [54, 63]. IC devices are not resistant to moisture, and therefore, humidity in the circuit connected to the mechanical ventilator should be prevented as much as possible by the use of the correct filters, pointing all sample lines upwards, postponing nebulization until after the measurement, and performing timely endotracheal suctioning (although as mentioned before, not within 20 min before measurement, to avoid agitation) [23].

A period of gas exchange in which VO_2_ and VCO_2_ vary by less than 5% over 5 min or 10% over 10 min should be chosen for calculations, although newer devices may do this automatically [8, 25, 80]. Measuring EE in spontaneously breathing and conscious patients could bring difficulties in accepting a canopy hood or face mask because of agitation, claustrophobia, or nausea [1, 61].

IC devices use various disposables at the patient circuit designed for one-time use only, such as flowmeters, filters, adapters, and sampling lines or, alternatively, a canopy hood, to ensure maximum hygiene. The device itself should be completely disinfected after each use. Nevertheless, connection of the IC device to a ventilation circuit requires a brief disconnection of the circuit, resulting in the release of aerosols. Therefore, care should be taken that connection of an IC device to the ventilation circuit of a patient with a disease that is transmittable through aerosols, such as COVID-19, is performed by personnel wearing protective garments and, when possible, takes place in a negative pressure room. Some guidelines advise against the use of IC in COVID-19 patients owing to potential aerosol exposure and therefore infection risk to healthcare providers [89], although others emphasize its value and offer practical guidelines to ensure optimal safety [90].

### Alternatives to indirect calorimetry

Several alternatives are used in research and clinical practice to estimate EE in situations where IC is not available or feasible.

#### Predictive equations to estimate energy expenditure

Predictive equations estimate a patient’s energy expenditure using anthropometry and vital parameters to estimate EE. All equations are unreliable, as EE is affected by many individual factors unaccounted for in the formulas [1, 7, 19, 31, 55, 91–95]. When comparing the results of predictive equations to those of IC, many discrepancies are found [18]. Consequently, the use of predictive equations alone is likely to lead to under- and overfeeding [51]. Nutritional guidelines discourage the use of these equations and advise never to administer more than 70% of the caloric need calculated based on these equations during the first week of ICU stay to prevent overfeeding [18, 22].

#### Ventilator VCO_2_ to estimate energy expenditure

Methods to calculate energy expenditure (EE) based on CO_2_ measurements (from the mechanical ventilator, or the pulmonary arterial catheter) have been proposed as a surrogate to IC. The EEVCO_2_-method uses VCO_2_ obtained from the mechanical ventilator or pulmonary artery catheter and a fixed RQ value of 0.86 to substitute VO_2_, for mechanically ventilated critically ill patients based on the enteral nutritional products most used in the ICU setting [96]:

$$ \mathrm{RQ}={\mathrm{VCO}}_2/{\mathrm{VO}}_2 $$

$$ {\mathrm{VO}}_2={\mathrm{VCO}}_2/\mathrm{RQ},\mathrm{with}\ \mathrm{RQ}=0.86 $$

The Weir’s equation is then adjusted as follows [18, 96]:

EEVCO_2_ (kcal/day) = 1.44 × (3.941 × [VCO_2_(mL/min)/0.86] +   1.11 × VCO_2_(mL/ min )), simplified:

$$ {\mathrm{EEVCO}}_2\ \left(\mathrm{kcal}/\mathrm{day}\right)={\mathrm{VCO}}_2\ \left(\mathrm{mL}/\min \right)\times 8.19 $$

Still, the use of a fixed RQ may lead to inaccuracies because of fluctuating substrate use. Applying the food quotient (FQ), or nutritional RQ, instead, may, in part, solve this inaccuracy [19, 30]. The approach assumes that the RQ value is equal to the FQ, i.e., the estimated RQs resulting from the oxidation of different energy substrates from nutrition therapy and non-nutritional calorie sources. The RQ is 1.0, 0.7, and 0.8 for carbohydrates, fat, and protein, respectively, enabling calculation of an individual FQ based on the composition of the administered energy sources (both nutritious and non-nutritious):

$$ \mathrm{FQ}=\left[\mathrm{fat}\%\times \kern0.37em 0.7\right]+\left[\mathrm{protein}\%\times \kern0.37em 0.8\right]+\left[\mathrm{carbohydrates}\%\times \kern0.37em 1.0\right] $$

Whenever relevant, other energy sources with different RQs can be added to the formula, such as in the case of citrate CVVH, where RQ_citrate_ = 1.33. Subsequently, the estimated RQ in the adjusted Weir’s equation is substituted for the calculated FQ. Nevertheless, the use of the FQs may be considered unreliable in patients in a catabolic state, as endogenous substrate utilization cannot be estimated by intake. In addition, the EEVCO_2_ method has consistently been shown to be inferior to IC [97, 98]. However, the technique has been proven to be more accurate than predictive equations [18, 91, 96].

## IC-guided nutrition

Despite guideline recommendations to use IC in critically ill patients, the superiority of IC-guided nutritional therapy has not yet been unequivocally proven in randomized clinical trials [15, 86, 99]. Even though it was confirmed that IC-guided nutrition support improves a patient’s nutritional status, the only significant benefit to outcome proven by RCTs is a significant decrease of nosocomial infections [46, 100–102]. Controversy exists concerning its effect on morbidity, mortality, and the length of hospital stay [18].

### Associations with clinical outcome

The pilot Tight Calorie Control Study (TICACOS) [29] suggested a 60-day mortality improvement in patients receiving higher caloric IC-guided nutrition than standard care, despite an increased length of ventilation and ICU-stay seen in this group. The subsequent TICACOS international study [103] showed that the use of an IC-guided nutritional goal yielded higher energy and protein delivery, compared with a nutritional goal based on predictive equations, with a trend toward lower mortality. However, overall results were insignificant. Covering 100% of repeated IC-derived REE from the first day of ICU in the EAT-ICU trial [104] did not affect the physical quality of life, infectious complications, or mortality at 6 months as compared to standard nutrition.

There are several possible explanations for these discrepancies. In the EAT-ICU trial, the defined nutritional goal of both protein and calories was largely met; however, the target was set only according to a median of two measurements per patient. For some patients, this meant that energy prescriptions were stationary after extubation, possibly underfeeding some at this stage. Conversely, aiming at covering 100% of measured REE in the early phase might have conferred overfeeding by exogenous nutrient overload in a phase when the endogenous substrate is mostly sufficient to meet REE. Zusman et al. [14] describe a U-shaped curve correlation between the percentage of calories delivered compared to measured EE and mortality in ICU patients, where both under- and overfeeding have harmful effects, and the beneficial effect lies in the middle of the curve. Therefore, caloric outliers on opposing sides of the curve might dilute any significant beneficial results. These observations further underline the need for studies addressing the effect of personalized IC-guided nutrition therapy based on repeat measurements, continued through various metabolic phases of illness and convalescence.

The question remains whether calories delivered to patients during the acute phase of their critical illness should match measured or estimated EE despite the ongoing endogenous nutrient release, which is not suppressed by feeding and remains immeasurable [85]. Furthermore, the effect of non-nutritional calories, including propofol, glucose, and citrate, should be taken into account when determining the target exogenous energy dosage [17]. Nutrition guidelines recommend to gradually advance to target during the first week, not meeting REE before the first 48 h to avoid overfeeding [18, 22].

An additional complexity in the interpretation of nutritional trials is the varied amount of protein delivered. The TITACOS studies were not protein targeted, and the amount of protein was determined by the rate of EN or parenteral nutrition provided. This resulted in patients receiving protein below the recommended levels. Current nutritional theory hypothesizes that not the caloric value, but the amount and timing of protein provided is most essential to influence the course of the disease, although the effect might not be the same in all types of critical illness [18, 105–108]. Therefore, results might reflect caloric overfeeding, early protein overdosing, late protein underfeeding, or a combination of these aspects. Future research should address the optimal timing and dosing of protein and calories individually.

### Respiratory quotient

Aside from energy expenditure, the IC derived RQ provides several theoretical applications, as the RQ indicates which macronutrients is mainly being metabolized. Underfeeding, which promote the use of endogenous fat stores, decreases the RQ, whereas carbohydrate metabolism increases RQ. However, studies in both adult ICU and pediatric burn patients found low sensitivity and specificity of IC derived RQ as an indicator of over- or underfeeding [60, 109]. Nevertheless, McClave et al. did show that increases in RQ correlated to increasing respiratory rate and decreasing tidal volume, suggesting that patients developed shallow, rapid respirations in response to increases in the measured overall RQ. Indeed lowering dietary fat guided by RQ can decrease VCO_2_ and thereby breathing effort in patients with obstructive lung disease, although the applications in the ICU setting are limited. More recently, several smaller studies found a correlation between (course of) RQ and outcome in critically ill patients, suggesting a potential prognostic use of RQ [110, 111]. Nevertheless, even if these patterns of substrate utilization could be reliably identified in larger populations, it remains unclear whether they can and should be influenced to improve outcome. Due to paucity of guiding evidence, it is currently advised that the clinical use of RQ is restricted to a marker of test validity to confirm measured RQ values are in physiologic range, and perhaps a rough estimation of respiratory tolerance of feeding [60].

All taken into account, the association of IC use with important clinical outcomes needs to be further explored before definitive conclusions about its use in the intensive care unit can be drawn. A recent systematic review and meta-analysis by Tatucu-Babet et al. [99] identified 4060 articles on the effect of IC-guided nutrition and clinical outcomes and found only 4 single-center, randomized controlled trials with 396 patients included in the analysis. All 4 studies reported higher receipt of energy close to the measured energy expenditure by IC compared to the predictive equation arm. However, when combined, no association between IC-guided energy delivery and hospital mortality was found, leading the authors to conclude that it is yet too early for widespread implementation of IC in clinical practice.

### Convalescence

No formal guidelines on calories and protein intake are available for the convalescence phase of critical illness. However, as patients likely enter a more physically active and anabolic phase with an increased TEE, it is assumed that a significant protein and calorie delivery is necessary to restore muscle mass and quality of life [17, 50]. Furthermore, studies imply that nutrition delivery largely fails to reach nutritional goals in the post-ICU hospitalization phase, although very few studies set goals according to regular IC measurements [11]. Recent retrospective data shows that PICS patients are prone to worse long-term outcomes and lower survival when fed with current evidence-based protocol nutrition [53]. It has been suggested that high levels of protein, amino acids, and anabolic adjuncts such as insulin, might aid in overcoming anabolic resistance in PICS. This is primarily extrapolated from cancer cachexia and burns research, and mechanistic studies are lacking [112, 113]. There is an urgent need for prospective studies measuring EE in the recovering critically ill and analyzing actual nutrition delivery and the effect on long-term outcome in different metabolic phenotypes.

## Conclusions

Energy expenditure appears highly variable among critically ill patients and in individual patients during various phases of illness. As a consequence, critically ill patients are at considerable risk of under- or overfeeding during ICU and post-ICU hospital stay, when rough and static estimates are used. The most recent international guidelines recommend regular indirect calorimetry to measure energy expenditure as a proxy for caloric requirement in ICU patients. However, the superiority of IC-guided nutritional therapy has not yet been unequivocally proven in randomized clinical trials and further research is urgently warranted. Nevertheless, IC has strong theoretical potential to improve nutritional status and consequently, the long-term outcome of critically ill patients in the various metabolic phases of critical illness. Increased knowledge of practical use and theoretical benefits of IC among clinicians can contribute to more widespread and routine use, thereby promoting research opportunities and real-time targeted and personalized nutrition therapy.

## Data Availability

Not applicable.

## Abbreviations

AEE: Activity energy expenditure
ATP: Adenosine triphosphate
BEE: Basal energy expenditure
BMR: Basal metabolic rate
CO2: Carbon dioxide
CRRT: Continuous renal replacement therapy
DIT: Diet-induced thermogenesis
ECMO: Extracorporeal membrane oxygenation
EE: Energy expenditure
FeCO_2_: Fractional concentration of carbon dioxide in expired air
FiO_2_: Fraction of inspired oxygen in expired air
IC: Indirect calorimetry
ICU: Intensive care unit
NO: Nitric oxide
O2: Oxygen
PEEP: Positive end expiratory pressure
PICS: Persistent inflammation, immunosuppression and catabolism syndrome
RCT: Randomized controlled trial
REE: Resting energy expenditure
RMR: Resting metabolic rate
RQ: Respiratory quotient
TEE: Total energy expenditure
TEF: Thermic effect of feeding
VCO_2_: Carbon dioxide transfer
VE: Volume of expired air
VI: Volume of inspired air
VO_2_: Oxygen transfer
